# The GA4GH Task Execution Application Programming Interface: Enabling Easy Multicloud Task Execution

**DOI:** 10.1109/mcse.2024.3414994

**Published:** 2024-06-19

**Authors:** Alexander Kanitz, Matthew H. McLoughlin, Liam Beckman, The GA4GH Cloud Workstream, Venkat S. Malladi, Kyle Ellrott

**Affiliations:** University of Basel, 4056, Basel, Switzerland; Microsoft Research and AI, Redmond, WA, 98052, USA; Oregon Health and Science University, Portland, OR, 97239, USA; Microsoft Research and AI, Redmond, WA, 98052, USA; Oregon Health and Science University, Portland, OR, 97239, USA

## Abstract

The Global Alliance for Genomics and Health (GA4GH) Task Execution Service (TES) application programming interface (API) is a standardized schema and API for describing and executing batch execution tasks. It provides a common way to submit and manage tasks to a variety of compute environments, including on-premises high-performance computing and high-throughput computing systems, cloud computing platforms, and hybrid environments. The TES API is designed to be flexible and extensible, allowing it to be adapted to a wide range of use cases, such as “bringing compute to the data” solutions for federated and distributed data analysis, or load balancing across multicloud infrastructures. This API has been adopted by numerous different service providers and is utilized by several workflow engines, yielding a single abstracted interface for developers and researchers. Using its capabilities, genome research institutes are building extensible hybrid compute systems to study life science.

In the field of bioinformatics and computational biology, and specifically the area of genomic computing, analysis of data is done by chaining together sets of command-line programs. There is no common programming language-level application programming interface (API) because each of these tools is the product of lengthy research in different areas of specialization written by different teams working at different institutions. These tools may be decades old or still in the development phase. Each of these tools may also have vastly different software stacks with different library dependencies. To deal with this situation, several groups have developed workflow engines that describe how these tasks are chained together. In conjunction with this, biological data are being generated by numerous different institutions and stored at service providers, including private institutional computing and commercial cloud providers. With the average Whole Genome Sequencing file being more than 200 GB, downloading all of the data to a single storage site is not practical. The Global Alliance for Genomics and Health (GA4GH) Task Execution Service (TES) API is used for describing and executing batch execution tasks, which are written to allow researchers to easily submit to and manage tasks on a variety of compute environments. GA4GH is an international community that seeks to advance human health by enabling the analysis of genomic and health-related data. A part of this effort includes the development of standards and frameworks to promote the secure, responsible, and effective use of genomic data. The TES API is one of the cloud-capable APIs that was developed by the GA4GH Cloud Workstream. The other APIs developed in this workstream include the Tool Registry Service (TRS), Workflow Execution Service (WES), and Data Registry Service (DRS) API specifications.

The TES API was designed to address on-premises high-performance computing/high-throughput computing (HPC/HTC) systems, cloud computing platforms, and hybrid environments. The TES API is designed to be flexible and extensible, allowing it to be adapted to a wide range of use cases. It can be used to execute simple tasks, such as running a single command on a single compute node, or as a back end for workflow management systems that are able to schedule the execution of individual tasks out of complex workflows involving multiple tasks and dependencies.

There have been limited previous efforts to standardize batch system interfaces, such as the Distributed Resource Management (DRM) API. This standard was developed in 2007 as an Open Grid Forum API specification for DRM systems, such as a cluster or grid computing infrastructure. Although supported by numerous HPC queuing systems, the specification was expressed as a C library, requiring software to have library-level bindings to access the API. Similarly, libraries such as the Portable Submission Interface for Jobs provide common language-specific bindings for accessing a variety of queueing engines. Each of these implementations are implemented as binding libraries and do not specify an interface for network-based clients. This is the major difference of the TES API: it is implemented from the start as a network-based API specification.

Globus Grid Resource Allocation and Management is a federated API for connecting to remote job schedulers. However, it is a single platform solution infrastructure and is not available to other systems outside of Globus. Kubernetes (frequently abbreviated as K8s) is an open source container orchestration platform widely used for managing containerized applications. Although Kubernetes is commonly employed in both cloud and on-premise environments, its primary concern is container orchestration through microservices. Although Kubernetes offers powerful orchestration capabilities, for batch computing, these capabilities are limited. The Kubernetes Jobs API does project capacity for batch execution; however, it does not manage large file transfer and is limited to Kubernetes-specific environments.

TES, on the other hand, was defined as an OpenAPI-defined specification for HTTP servers, based on the OpenAPI specification, allowing network access to the system, as well as lightweight library bindings to be rapidly generated for new programming languages. The flexibility of the TES API and its ability to be deployed in numerous different infrastructures is a valuable tool for the genomics community and other audiences that benefit from a cross-platform, cross-cloud batch execution solution. TES can help to simplify and streamline the execution of computational workflows. It can also help to reduce the cost of running these workflows by making it possible to use a variety of compute resources, including cloud computing platforms (see Figure 1). The ecosystem currently includes implementations that support HPC/HTC cluster-based job schedulers, such as SLURM and Grid Engine, Kubernetes-based systems and commercial cloud-specific APIs, including Amazon Web Services (AWS) Batch and Azure Batch. The API is designed to wrap various infrastructures and remove those complexities from the user’s concern. It can be deployed within a user’s environment on an HPC system, deployed at a project level within a cloud virtual private network, or presented as a gateway uniform resource locator (URL) for an entire organization. Multiple workflow engines have begun to utilize the API to deploy workloads on TES-fronted systems, including Galaxy,^1^ Cromwell,^2^ Nextflow,^3^ and Snakemake.^4^

## THE TES STANDARD

**FIGURE 2.**
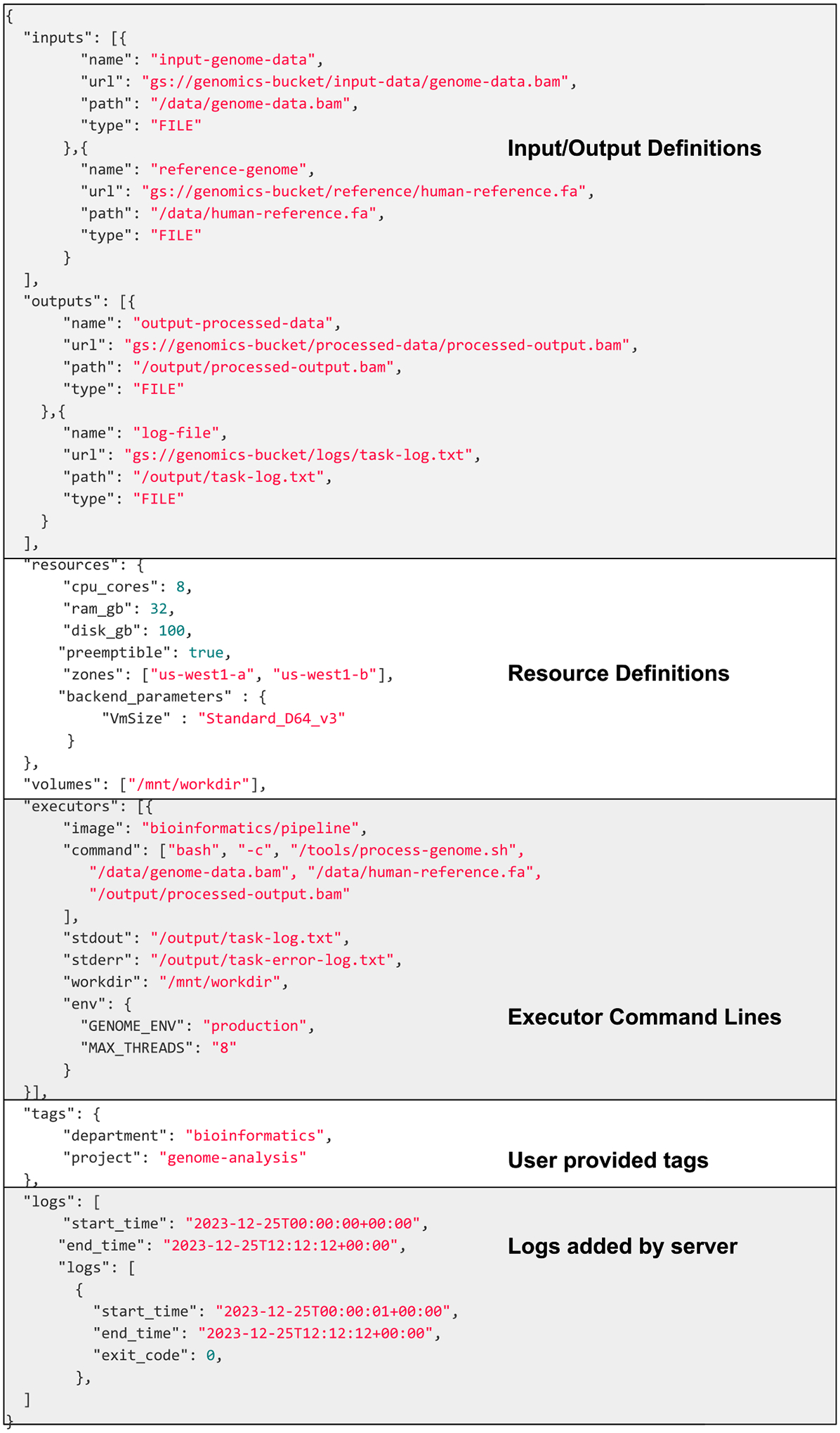
An example TES packet. The example TES task demonstrates the use of inputs, outputs, and logging.

At the core of the TES API is the idea that, for a new computational task to be issued into a compute environment, numerous core elements need to be defined so that systems can be adequately prepared for user code to be deployed. In a hybrid compute environment, there are numerous elements that must be explicitly defined to ensure that a command line is able to execute successfully. In many HPC/HTC environments, although a program command may be executed on an arbitrary node in the cluster, the system is usually set up in the exact same way as the submission host, including the same environmental variables, software libraries, and support programs. HPC/HTC environments will also usually have global access to a unified POSIX file system, e.g., via the Network File System (NFS). In a cloud environment, no such guarantees exist. A process may be initiated on a virtual machine that was just allocated and initialized based on a base operating system (OS) image with no additional software installed. Additionally, there is no guarantee that any particular file assets will be available, or that shared storage will be mounted. The core resource of the API is a task (see Figure 2), an atomic message that details all of the required task parameters. Because such guarantees are lacking in the execution environment, the TES specification requires numerous task parameters to be defined, including the following:

- **Application Environment**: User software and computational tools need to be packaged and deployed. As user jobs will be handled by potentially undefined systems, such as vanilla virtual machines, or HPC worker nodes with different OS versions and hardware architectures, the software required to run the job needs to be defined as part of the job. Software containerization systems, such as Docker images, have provided a mechanism to capture entire software environments, with all software dependencies, and rapidly deploy them on new computer systems.
- *Computational resources*: Tasks have defined hardware minimums that enable them to be run. This includes CPUs, GPUs, memory, and storage within a computing environment. It plays a crucial role in optimizing task execution by allocating resources dynamically based on task demands as provided in the API. The computational resources used for the job may be reclaimed and reset to be available for the next job.
- *Inputs*: Custom input files and directories for a specific task need to be transferred into place prior to the user task being invoked. These files need to be stored within a system that can be accessed by both the submission system and the eventual compute node. If all the processes are maintained within the same HPC environment, this can be represented using traditional network attached storage, such as an NFS instance. In the case of cloud environments, object storage systems, such as S3, can be used as the intermediate storage instead.
- *Outputs*: Next to requiring custom inputs, most jobs will create one or more output files and directories that need to be exported to a common storage system at the end of the job as any files not included in an export manifest may be lost after the end of a job.
- *Environmental variables*: A tool’s behavior may be modified based on the settings of environmental variables, such as the current working directory. TES therefore supports the definition of such variables.
- *Command lines*: The user process will need a command line to be executed in the environment set up by the TES service as a result of the information stated in this list. TES supports the definition of multiple command lines for each request, e.g., to define setup and tear-down jobs for the main job, or for the grouping of multiple command lines with similar requirements. When multiple command lines are defined in a TES request, they will be executed sequentially. Each command can utilize a different container image, but inputs, outputs, and volumes are shared across commands.

**FIGURE 3.**
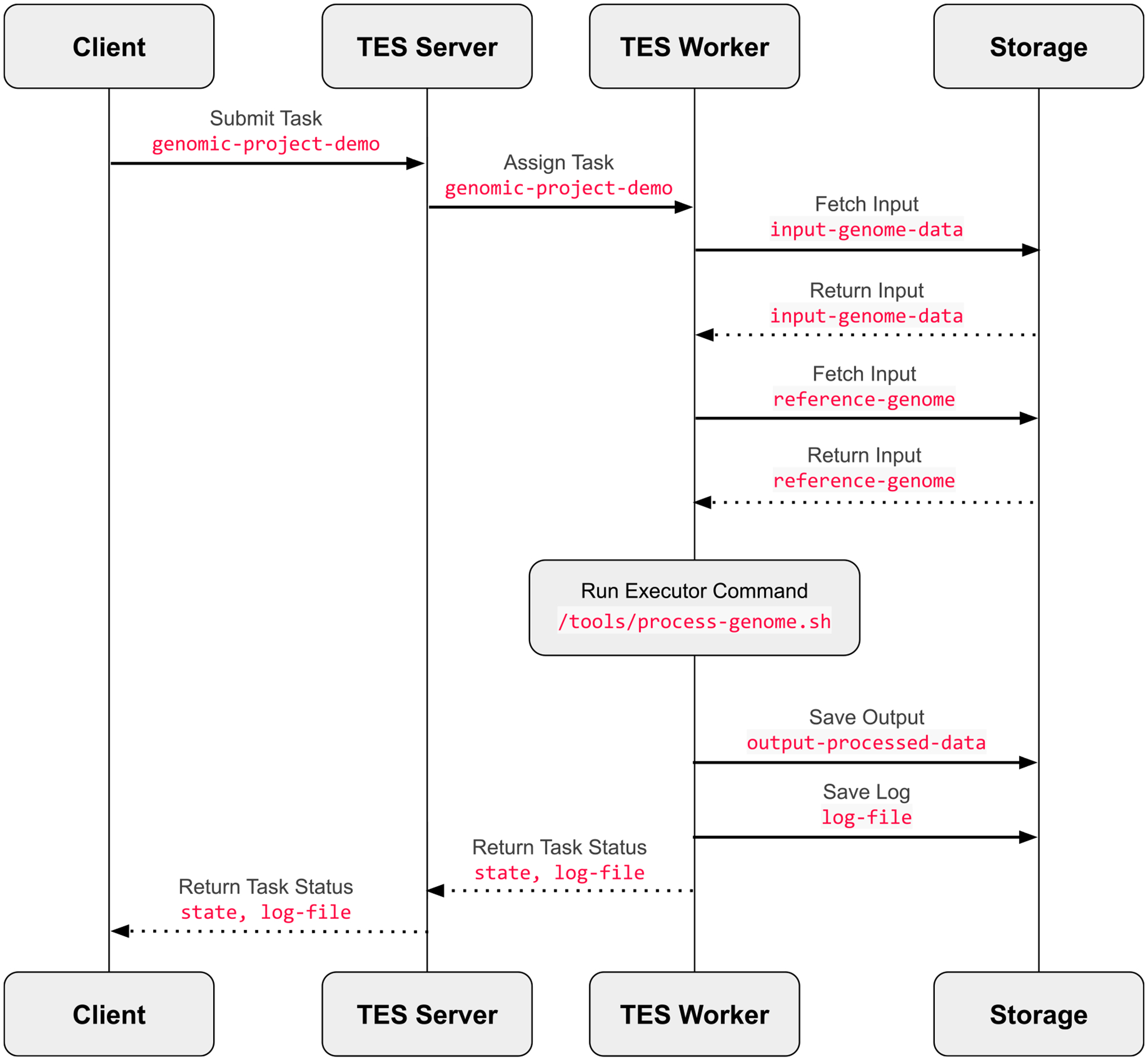
The TES execution architecture. The figure presents an outline of the separate layers found in current TES service implementations. The client talks to a server, which is responsible for allocating a worker node on an HPC/HTC or cloud infrastructure. The TES worker is responsible for transferring inputs, running user code, capturing logging, and storing outputs.

TES-compatible clients and servers can be developed and used independently. This makes it easy to integrate the TES API into existing workflow management systems and other software tools. Because the TES task messages are atomic, containing all the information needed to successfully complete a set of command lines, they may be easily passed among clients, servers, proxy servers, databases, and event streams. Given this definition of task, we have provided a framework for TES tasks that can be executed on a TES-compatible server, with the details of HPC/HTC versus the cloud compute environment abstracted away (see Figure 3). This makes it easy to port workflow processes to new compute resources. In practice, by providing this core API, the TES API is flexible enough to be used to execute a wide range of tasks, from simple commands to interacting with engines to orchestrate complex workflows.

The TES API is based around the creation, monitoring, controlling and listing of task messages. These have been analogized in the TES API as resources under the “/tasks” endpoint, with POST creating new resources and GET returning a paginated listing of available resources. Task resources are referenced by a unique identifier, which is assigned by the server and returned when a resource is created. Using the task identifier, it is also possible to cancel a given task (via POST/tasks/{id}:cancel) or retrieve detailed information on a task resource (via GET/tasks/{id}). Aside from requiring task identifiers to be unique for a given TES instance, the TES API does not impose any other restrictions on how these identifiers are formed.

The length of time for which tasks are stored is not prescribed by the TES specification and is up to the implementer or an individual TES instance’s configuration. When listing available resources, the API provides a mechanism to filter tasks by state or name prefix. When creating tasks, clients are allowed to attach an arbitrary set of key/value string pairs to a task, which can be used for storage of identifiers or any other metadata that clients deem important. These user-/client-defined tags may also be used to filter tasks. For the GET methods described in this section, the API allows the user to customize the level of detail for listing into three levels: MINIMAL, BASIC, and FULL.

FTP support in TES relies on external standards. Inputs and outputs are represented as arbitrary strings, which an implementation then tries to interpret as URLs according to the rules and conventions of supported protocols. To find out which protocols are supported by a given TES instance, clients can query the /service-info endpoint via the GET method. The storage property will list the supported FTPs. For example, a TES implementation supporting S3 buckets, FTP, and HTTP URLs might list s3, ftp, and http.

The way in which TES instances should set up a software environment to execute a command line also relies on arbitrary strings, supplied via the image property of a TES executor (see below Figure 2). It is currently expected that TES instances will support uniform resource identifiers that point to Docker images. Explicit support for other containerization frameworks, or even container-independent methods for specifying software environments (e.g., Conda), is planned.

Two key elements of the TES specification, file systems and software containerization, are reliant on external standards. The file system can be represented, for example, by a shared file system, an S3 bucket, or FTP HTTP URLs. For containerization, the TES API expects a Docker-formatted container that contains all the software and dependencies for a given job. In both cases, the corresponding properties of the TES schema allow for arbitrary strings to represent elements and an understanding of the capabilities of the TES server implementation.

File transfer is not a part of the specification to ensure that the TES server does not become a bottleneck in large file transfer. Many use cases of the TES API involve processing genomic files that are several hundred gigabytes large. It is expected that the user will stage these files in an appropriate file storage system; for example, when deploying on AWS, the files would be staged to an S3 bucket. The GA4GH DRS API manages translation of file identifiers into potential file sources, including enabling a system to identify the mirroring locations that are closest to where the computation will be deployed.

A TES task is allowed to have multiple command lines. In the TES API terminology, each command line is defined in an *executor*, and, when sending a task request, a client is able to specify multiple executors (specifically, the corresponding executors property accepts an array of executors) in an array of “executors.” Each executor defines the command line, container image, working directory, stdin/stderr mapping, and environmental variables. Executors are run sequentially, one at a time. If the ignore_error flag is not set (default), a nonzero exit code from one of the executors (i.e., command-line invocations) will cause the sequence to end early. Although this mechanism could, in principle, be employed to schedule the execution of linear subworkflows of a given workflow on the same TES instance for optimization purposes, this was not the primary intent. Rather, the ability to supply multiple instructions for a given task resource provides an opportunity for clients to drop in setup and finalization methods on the worker node, e.g., for increased compatibility with existing workflow management system designs.

To bridge a crucial gap in the adoption of the TES standard, the TES community recently created a conformance test suite for the GA4GH TES API.^5^ The tests are defined based on a YAML-based specification that a dedicated test runner knows how to interpret. This extensible test suite ensures that developers can confidently build tools and services that adhere to the API specification, both when developing new implementations and when upgrading existing implementations to support new versions of the specification. Currently available conformance tests cover various aspects of the TES API, including data structures, messaging protocols, and some functional requirements. The testing framework, consisting of the test runner, the test specification, and the actual test suite, employs a modular design, allowing for targeted testing of specific API components, or the entire API in one go.

TO BRIDGE A CRUCIAL GAP IN THE ADOPTION OF THE TES STANDARD, THE TES COMMUNITY RECENTLY CREATED A CONFORMANCE TEST SUITE FOR THE GA4GH TES API.

## ECOSYSTEM

The goal of TES is to provide an open specification that different groups can utilize, and to create a robust ecosystem of services that provide software platforms that utilize it. Ideally, a researcher should be able to easily move from HPC to any of the cloud vendors and not need to change this workflow management system. We measure the success of that goal by the number of different implementations that are available and compatible with the API conformance suite (see Table 1).

The first element of a robust API ecosystem would be the service providers that implement TES. There are already four service providers that officially provide the API to different types of underlying infrastructures.

The Microsoft GA4GH-TES^6^ on Azure project provides a TES API implementation for Microsoft Azure, backed by the Azure Batch service. This C# implementation is one of the core technologies being used to implement the Cromwell engine and Broad’s Terra platform on Azure. Funnel^7^ is an implementation of the TES API, designed to run tasks on various computing environments such as Slurm, HTCondor, Google Compute Engine, AWS Batch, and others. It was developed by a team at Oregon Health and Science University. TESK^8^ is a Kubernetes-native free and open source implementation of the TES API, originally developed by the European Bioinformatics Institute of the European Molecular Biology Laboratory (EMBL-EBI), and now maintained by the ELIXIR Cloud and authentication and authorization infrastructure (AAI) GA4GH Driver Project. TESK acts as a batch-scheduling system for Kubernetes and compatible Native Cloud solutions like OpenShift. Pulsar is a server platform for running jobs from the Galaxy workflow engine on remote systems. They have added support for the TES API so that the additional clients that use TES can plug in to their network of deployed sites.

Beyond servers that provide the TES API, there is a potential for meta services that are able to provide flexible middleware injections to effectively federate atomic, containerized workloads across various environments. For example, proTES is a scalable gateway service designed to offer centralized features to a federated network of TES instances, such as serving as a compatibility layer for different TES implementations, a head node for smart workload distribution across the network, a public entry point to an enclave of private TES nodes, or a means of collecting telemetry. A plug-in system allows for the easy creation and injection of middleware tailored to specific use cases and requirements, such as access control, request/response processing, or the selection of suitable TES endpoints considering legal constraints (e.g., data use) and user/client preferences.

The final element of the ecosystem is the client software that utilizes the API. Numerous workflow orchestration engines have started to utilize TES as a way to issue required commands as they manage task dependencies. Cromwell is an open workflow engine that was originally developed by the Broad Institute for the orchestration of bioinformatic workflows. It’s one of several tools and platforms that are able to run workflows written in the Workflow Description Language. Snakemake is a Python-based workflow management system designed for reproducible and scalable data analysis that utilizes the TES API to enhance its capabilities. To better support the execution of Snakemake workflows on various compute back ends, Snakemake developers have developed a plug-in system for such executors for their recent 8.0 release. One such plug-in is specifically designed for submitting jobs via the TES API. Nextflow, a groovy-based workflow management system designed for portability and scalability, has long supported the GA4GH TES API to enable flexible and adaptable task execution across diverse computing environments.^3^ The limitations that have prevented a more widespread adoption of the TES back end in Nextflow have been addressed on the side of the specification with the release of TES v1.1.0. In the Nextflow workflow management system, developers are currently working toward implementing these changes toward a more powerful TES back end. For the Common Workflow Language (CWL),^9^ cwl-tes is an experimental workflow engine based on the CWL reference engine that is specifically built around a TES executor. For the Toil^10^ workflow management system, which also supports the execution of CWL workflows, a prototype TES back end has recently been added. Next to workflow management systems, the TES client ecosystem also includes the Python library py-tes for accessing TES servers programmatically (the library is used in some of the workflow management system back ends, e.g., the Snakemake one). Finally, a reusable Web Component-based micro front end for the TES API is currently being developed as part of the ELIXIR Cloud Components suite.^11^

The list of publicly available workflow engines is constantly growing. They include entries such as Air-flow, Arvados, Pegasus, Luigi, MiniWDL, GoFlow, Kube-Flow, Yet Another Workflow Language (YAWL), and hundreds of others. At their base, each of these systems needs to build wrappers for deploying asynchronous tasks in different environments. By utilizing the TES API each of these systems would be able to deploy jobs in HPC and multiple cloud environments using a single API.

## DISCUSSION

TES’s main goal is to promote interoperability and to bring together different computational software platforms and infrastructure. Consistent with this goal, the ELIXIR Cloud and AAI GA4GH Driver Project^12^ is prototyping a cloud infrastructure that uses the TES API at its core to federate the execution of computational workflows across different national nodes in the ELIXIR community. Similarly, the European Genomic Data Infrastructure, an ambitious project aiming to connect institutions from 20 different European countries for the federated analysis of genomics data, from hospitals to research centers, has built their federated compute capabilities around the TES API into their Starter Kit. Microsoft initially utilized TES as a way to support the Cromwell workflow engine to work on Azure. The implementation was initially rolled into the CromwellOnAzure deployment. With further development, use cases, interest from the community, and other workflow engines, the TES implementation was split into its own stand-alone repository and deployment.^6^

The API’s design philosophy is to maintain a lightweight interface that allows for multiple implementers to easily build compliant clients and servers. The API will continue to evolve, but only in a way that maintains this lightweight interface design. In many cases, evolution of the API will not involve changes to the OpenAPI description, but rather further refinements to the conformance test suite. One of the main goals of future versions is to provide better guidance around authentication, security, and software portability. The way that credentials and authorization flow through the various layers of the infrastructure is very important to maintain interoperability of different solutions, particularly in multicloud applications. Passing storage credentials from the user to the node, where execution of the job will occur, needs to be outlined. Additionally, a generalized solution that can pass compute authorizations from users to API endpoints as well as how to restrict compute available to users in hybrid systems, the cloud, and HPC/HTC, needs to be further developed.

The TES API was originally designed around Docker, with the presumption that the image definition would map to the image name passed to a Docker command line. However, as implementations and use cases evolved, it became clear that other containerization systems, such as LXC, Singularity, rkt, and Podman, would also be seen as possible use cases of security and architecture. The TES deployment can utilize any containerization technology that is applicable; for example, in HPC environments, user-level containerization systems such as Apptainer and Podman are preferable for security. The TES server is required to do any image translation from an Open Container Initiative Image Format to deployment formats, such as Singularity Image File automatically. The end user of the API should be unaware of the underlying containerization system deployed on the system.

There has been discussion around possible extensions to the API. Ideas such as enabling privacy-preserving federated learning, enabling Hadoop or Spark jobs, supporting federated learning use cases, and a callback API have all been suggested as possible developments for the TES API. These extensions could be added to the API as optional extensions that would not be required for basic conformance.

The TES API has been successfully utilized to submit arrays of 10,000 jobs and has managed sets of several thousand cloud virtual machines. The scalability of the TES API is largely dependent on the underlying platform that implements the specification. When deploying in a public cloud environment, the scaling capacity will largely be determined by the infrastructure speed of the cloud provider and capacity. Although in an HPC environment, it will be dictated by the resource available on the queue.

## CONCLUSION

The GA4GH Cloud Workstream has been working and developing numerous cloud-integration APIs, including the TRS, WES, and DRS. These APIs cover the location of datasets and tools and communication with workflow engines. The need for a low-level API that allowed task-deployment systems to move among compute environments was identified as a blocker to enabling cloud interoperability and hybrid computing. The TES API was proposed and developed over several years by working with community members and driver projects. Various suggestions were considered for inclusion, and the specification has been refined to address gaps found when potential collaborators evaluated the system. The 1.0 version of the specification was accepted by the GA4GH Standards Steering Committee in 2021. The 1.1 update was released roughly a year later to address the gaps found by driver projects.

The TES API is designed to be interoperable, meaning that TES-compatible clients and servers can be developed and used independently. This makes it easy to integrate the TES API into existing cloud infrastructures, workflow management systems, and other relevant software solutions. Importantly, this API allows researchers to develop their analysis workflows independent of their infrastructure and deploy them in a multicloud approach, targeting computational resources that are more affordable or that are closer to the data that need to be processed. We have already seen a large uptick in the number of groups utilizing the TES API to power their systems, both in academia and industry, thus demonstrating its utility in powering computational analysis for life sciences.

## Figures and Tables

**FIGURE 1.**
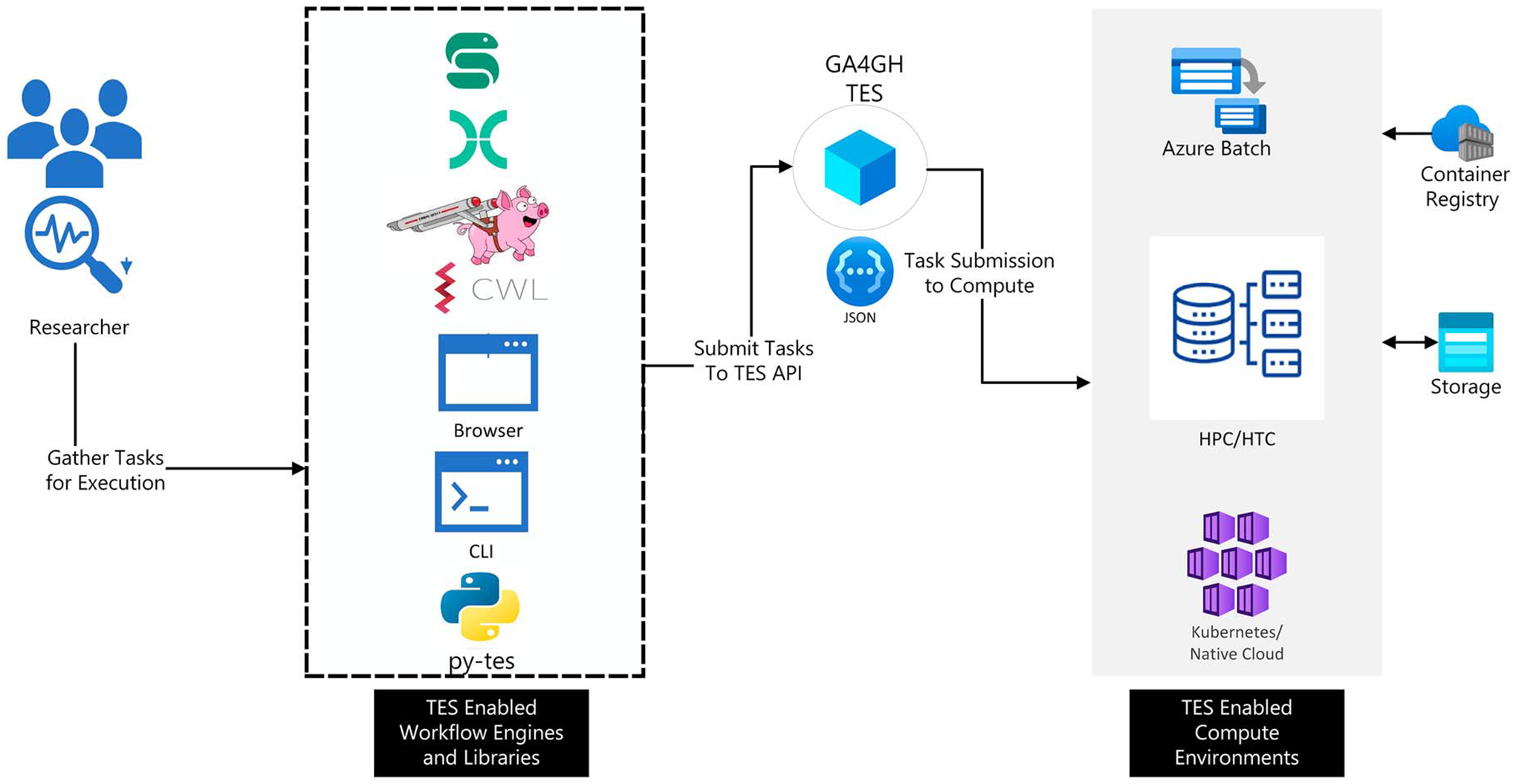
Common TES use cases. The TES API wraps around compute environments providing a standard way of executing tasks. Researchers can write and package their tasks and data in a domain-specific language (DSL) workflow language. They then hand the orchestration of the tasks over to the respective workflow management systems. The workflow management systems can then make use of TES clients to distribute tasks across different environments. Alternatively, users can submit individual tasks to TES servers directly via command-line interfaces (CLIs) or GUI interfaces. Thus, TES makes it easier for researchers to make use of a variety of compute environments seamlessly. Applications can support new compute environments by integrating with TES APIs, rather than develop unique connections for each environment. CWL: Common Workflow Language.

**TABLE 1.** The TES ecosystem. The table lists the available servers, proxy, and client implementations that utilize the TES API.

Type	Project	Description	Source
API	TES	OpenAPI definition of the specification	github.com/ga4gh/task-execution-schemas
API	TES	Conformance test runner	github.com/elixir-cloud-aai/openapi-test-runner
Server	Funnel	TES server implementation for HPC/HTS systems, including AWS Batch, Google Cloud, Kubernetes, Slurm, GridEngine, and HTCondor	github.com/ohsu-comp-bio/funnel
Server	Pulsar	TES server implementation for the Galaxy/Pulsar federated distributed network	pulsar.readthedocs.io
Server	TES-Azure	TES server implementation for the Microsoft Azure	github.com/microsoft/ga4gh-tes
Server	TESK	TES server implementation for Kubernetes/Native Cloud systems	github.com/elixir-cloud-aai/TESK
Proxy	proTES	Proxy service for injecting middleware into GA4GH TeS requests	github.com/elixir-cloud-aai/proTES
Client	Cromwell	Workflow management system for executing workflows composed in the Workflow Definition Language domain-specific language (DSL)	cromwell.readthedocs.io
Client	cwl-tes	Workflow management system for executing workflows in the Common Workflow Language (CWL) DSL	github.com/ohsu-comp-bio/cwl-tes
Client	ELIXIR Cloud Components	Web Component library for interacting with TES services (and other GA4GH APIs)	elixir-cloud-components.vercel.app
Client	Nextflow	Workflow management system for executing workflows composed in the Nextflow DSL	nextflow.io
Client	py-tes	Python client library for interacting with TES services	github.com/ohsu-comp-bio/py-tes
Client	Snakemake	Workflow management system for executing workflows composed in the Snakemake DSL	snakemake.github.io
Client	Toil	Workflow management system for executing workflows composed in the Toil and CWL DSLs	toil.readthedocs.io
